# Macrophage LRP1 Suppresses Neo-Intima Formation during Vascular Remodeling by Modulating the TGF-β Signaling Pathway

**DOI:** 10.1371/journal.pone.0028846

**Published:** 2011-12-09

**Authors:** Selen Catania Muratoglu, Shani Belgrave, Anna P. Lillis, Mary Migliorini, Susan Robinson, Elizabeth Smith, Li Zhang, Dudley K. Strickland

**Affiliations:** 1 Center for Vascular and Inflammatory Diseases, School of Medicine, University of Maryland, Baltimore, Maryland, United States of America; 2 Department of Physiology, School of Medicine, University of Maryland, Baltimore, Maryland, United States of America; 3 Department of Surgery, School of Medicine, University of Maryland, Baltimore, Maryland, United States of America; 4 Department of Pathology, Duke University Medical Center, Durham, North Carolina, United States of America; Leiden University Medical Center, The Netherlands

## Abstract

**Background:**

Vascular remodeling in response to alterations in blood flow has been shown to modulate the formation of neo-intima. This process results from a proliferative response of vascular smooth muscle cells and is influenced by macrophages, which potentiate the development of the intima. The LDL receptor-related protein 1 (LRP1) is a large endocytic and signaling receptor that recognizes a number of ligands including apoE-containing lipoproteins, proteases and protease-inhibitor complexes. Macrophage LRP1 is known to influence the development of atherosclerosis, but its role in vascular remodeling has not been investigated.

**Methodology/Principal Findings:**

To define the contribution of macrophage LRP1 to vascular remodeling, we generated macrophage specific LRP1-deficient mice (macLRP1-/-) on an LDL receptor (LDLr) knock-out background. Using a carotid ligation model, we detected a 2-fold increase in neointimal thickening and a 2-fold increase in the intima/media ratio in macLRP1-/- mice. Quantitative RT-PCR arrays of the remodeled vessel wall identified increases in mRNA levels of the *TGF-β2* gene as well as the *Pdgfa* gene in macLRP1-/- mice which could account for the alterations in vascular remodeling. Immunohistochemistry analysis revealed increased activation of the TGF-β signaling pathway in macLRP1-/- mice. Further, we observed that LRP1 binds TGF-β2 and macrophages lacking LRP1 accumulate twice as much TGF-β2 in conditioned media. Finally, TNF-α modulation of the *TGF-β2* gene in macrophages is attenuated when LRP1 is expressed. Together, the data reveal that LRP1 modulates both the expression and protein levels of TGF-β2 in macrophages.

**Conclusions/Significance:**

Our data demonstrate that macrophage LRP1 protects the vasculature by limiting remodeling events associated with flow. This appears to occur by the ability of macrophage LRP1 to reduce TGF-β2 protein levels and to attenuate expression of the *TGF-β2* gene resulting in suppression of the TGF-β signaling pathway.

## Introduction

The LDL receptor-related protein 1 (LRP1) is a large endocytic receptor that was initially identified as an hepatic receptor for α_2_-macroglobulin complexes [1]–[3]. Subsequent work has revealed that LRP1 recognizes numerous ligands and contributes to a variety of cellular functions and signaling events [4], [5]. Within the vasculature, LRP1 appears to play a protective role. Thus, generation of an LRP1 knock-in mouse with mutations in a critical NPxYxxL motif within its cytoplasmic domain resulted in increased atherosclerosis when crossed into an LDLr-deficient background [6], revealing that impaired function of LRP1 alters the progression of this disease. Further, hepatic deletion of LRP1 also led to increased atherosclerosis indicating that hepatic LRP1 function also regulates the development of atherosclerosis. Mice with LRP1 genetically deleted in vascular smooth muscle cells display excessive activation of the PDGF signaling pathway resulting from increased expression of the PDGFR in the vessel wall [7] demonstrating that in smooth muscle cells, LRP1 protects the vasculature by suppressing the excessive activity of this pathway. Deletion of LRP1 within macrophages has been shown to enhance the extent of atherosclerosis in LDL receptor/apoE double knockout mice [8] and in LDL receptor knockout mice receiving a bone marrow transplant from mice in which LRP1 was selectively deleted in macrophages [9]. Currently, the mechanism by which macrophage LRP1 impairs lesion development in atherosclerosis is not understood.

In addition to their contribution to the development of atherosclerosis, macrophages are also known to contribute to restenosis. Restenosis and in-stent restenosis occurs following percutaneous balloon angioplasty, an established method for treating severe coronary artery blockage [10]. Restenosis involves significant vascular remodeling including excessive deposition of matrix proteins, as well as migration and proliferation of vascular smooth muscle cells. In response to injury, these cells de-differentiate from a quiescent, differentiated state to a proliferating and synthetic phenotype [11]. Major contributors to these processes are the PDGF [12] and TGF-β [13] signaling pathways. To determine if macrophage LRP1 modulates vascular remodeling during restenosis and to gain mechanistic insight into these processes, we initiated studies comparing macrophage-deleted LRP1 mice (macLRP1-/-) to control mice expressing LRP1 in an established model of carotid artery ligation [14]. Our results reveal that macrophage LRP1 suppresses neointima formation, and further implicate a mechanism in which LRP1 modulates the TGF-β signaling pathway.

## Results

### Genetic deletion of LRP1 in macrophages increases intimal hyperplasia following carotid artery ligation

**Figure 1:**
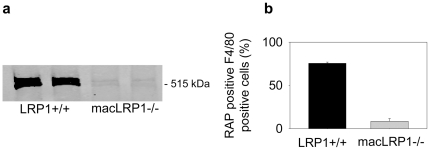
Effective deletion of the *Lrp1* gene in macrophages from macLRP1-/- mice. a) Bone marrow derived macrophages from LRP1+/+ and macLRP1-/- mice were analyzed for LRP1 expression by immunoblot analysis. b) Resident macrophages were isolated by peritoneal lavage and incubated with fluorescent labeled RAP for 1 h at 37 ^o^C. RAP-positive, F4/80 positive cells were then determined by FACS analysis.

To evaluate the contribution of macrophage LRP1 to vascular remodeling, we employed the well characterized carotid artery ligation model [14]. The contribution of macrophages to arterial wall remodeling is well established [15], [16] and occurs early in this model. Further, macrophage contribution is significantly enhanced in apoE- or LDL receptor (LDLr)-deficient mice maintained on a high fat diet [15], and thus we generated a macrophage specific LRP1 knock-out mouse on an LDLr-/- background (termed, macLRP1-/-) and evaluated vascular remodeling when the mice were maintained on a high fat, Western-type diet. To evaluate the effectiveness of the genetic deletion, bone marrow derived macrophages from LRP1+/+ and macLRP1-/- mice were subjected to immunoblot analysis. The results (Fig. 1a) reveal effective deletion of LRP1 in macrophages from the macLRP1-/- mice consistent with our previous results [17]. LRP1 function was also ablated in resident macrophages obtained by peritoneal lavage. This was confirmed by measuring the ability of cells staining positive for the macrophage marker F4/80 from macLRP1-/- mice to internalize fluorescent-labeled receptor associated protein (RAP) which binds tightly to LRP1 (Fig. 1b). Together, these results reveal an effective ablation of LRP1 antigen in bone-marrow derived and resident macrophages in the macLRP1-/- mice.

**Figure 2:**
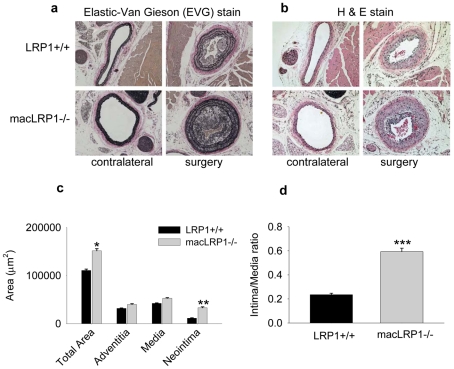
LRP1 expressed in macrophages protects against vascular remodeling in the carotid-ligation model. a) Photomicrographs showing the representative Elastic-Van Gieson staining of contralateral (*left panels*) and ligated vessels (*right panels*) in LRP1+/+ (*top*) and macLRP1-/- (*bottom*) mice. b) H&E staining of the serial sections of the same artery as in (a). c) Morphometric measurements of the total vessel area, adventitia area, media area and intima area were measured for LRP1+/+ (n = 20) and macLRP1-/- (n = 20) mice (*p = 0.03; **p = 0.009, Students *t*-test). d) Ratio of the Intima over the Media is represented for LRP1+/+ and macLRP1-/- mice (***p = 0.010, Students *t*-test).

No morphological differences were noted in the carotid arteries of untreated mice when macLRP1-/- mice were compared with control mice (LRP1+/+, also on an LDLr-/- background). In contrast, two weeks following ligation with the mice maintained on a Western diet, we detected much more extensive remodeling in the macLRP1-/- mice than noted in LRP1+/+ sibling mice (Fig. 2). We stained consecutive sections with elastic-Van Gieson (EVG) (Fig. 2a) to detect the elastic lamina and H&E (Fig. 2b) to detect the cellular composition of the neointima. These data revealed that carotid arteries from both LRP1+/+ and macLRP1-/- mice have a highly cellular neointima (Fig. 2b). Remodeling was measured by morphometric analyses of the intimal area and by calculating the ratio of intimal area to medial area which were determined on EVG stained paraffin sections. These analyses revealed a 2.4-fold increase in neointimal thickening and in the intima/media ratio in macLRP1-/- vessels compared to vessels from LRP1+/+ mice (Fig. 2c,d). In addition, the overall area of the vessels was slightly larger in the macLRP1-/- mice.

**Figure 3:**
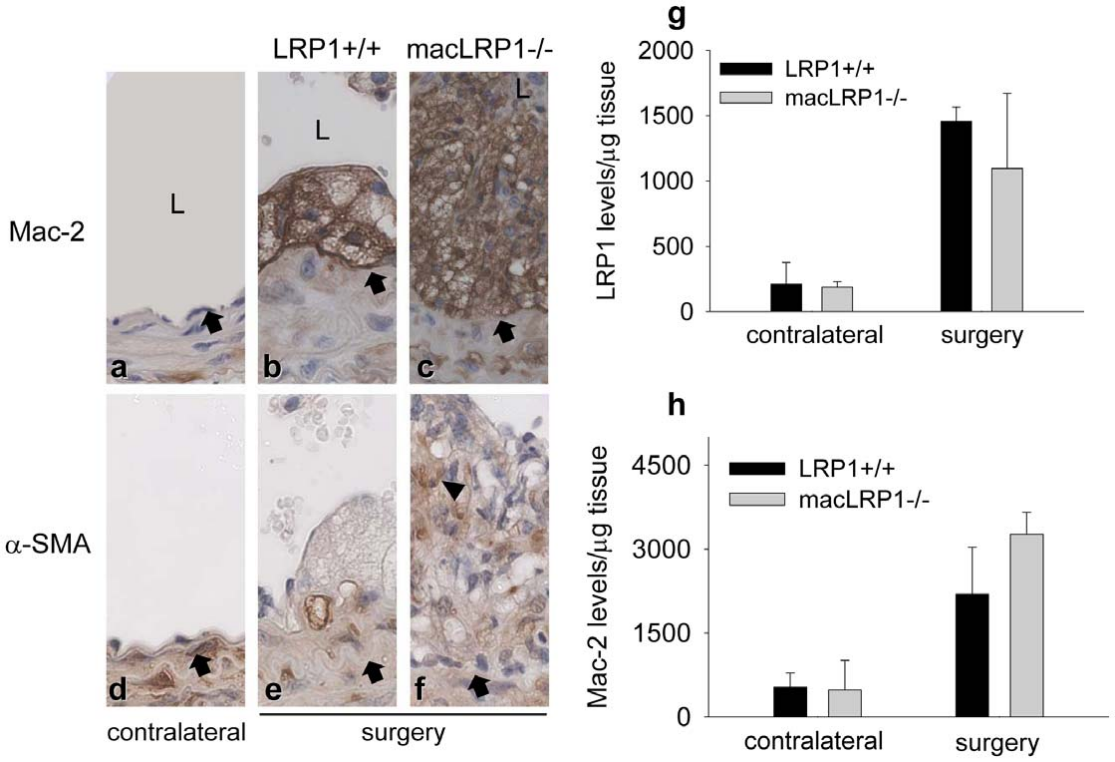
Immunohistochemical analysis of macrophage and α-SMC actin positive cells in the neointima. Cross-sections of contralateral (a,d) and ligated (b,c,e,f) carotid arteries 2 weeks after ligation. Representative expression of Mac-2 (a,b,c) and α-SMA (d,e,f) antigen in contralateral and ligated vessels. Internal elastic lamina (IEL) is marked by a black arrow. Arrowhead indicates the cells positive for antibody staining. L shows lumen. g) Immunoblot analysis of LRP1 expression in contralateral and surgery vessels of LRP1+/+ (n = 2) and macLRP1-/- (n = 2) mice. Gels were analyzed using NIH imageJ h) Immunoblot analysis of Mac-2 expression in contralateral and ligated vessels of LRP1+/+ (n = 4) and macLRP1-/- (n = 4) mice. Gels were analyzed using NIH imageJ.

To identify the cell types involved in vascular remodeling that are present within the neointima, serial sections of both ligated and contralateral arteries were subjected to immunohistochemical analysis employing markers of macrophages (Mac-2/Gal3; soluble lactose binding lectin-2) (Fig. 3a, b, c) and smooth muscle cells (smooth muscle actin α-SMA) (Fig. 3d, e, f). These analyses revealed that the majority of neointimal cells in LRP1+/+ carotid arteries were macrophages (Fig. 3b) along with a relatively small number of α-SMA positive cells (Fig. 3e). In contrast, in the neointima of macLRP1-/- mice, there were a large number of α-SMA positive cells (Fig. 3f) as well as macrophages (Fig. 3c). In both LRP1+/+ and macLRP1-/- mice, the macrophages resemble foam cell macrophages, most likely resulting from the “Western diet”, and these cells are known to contribute significantly to vascular remodeling [15], [18]. We characterized the total expression of LRP1 and Mac-2 antigen in the arterial wall of LRP1+/+ and macLRP1-/- mice using immunoblot analysis, and the results revealed that the expression of LRP1 in vessels undergoing ligation were substantially increased (Fig. 3g), likely due to the increased accumulation of smooth muscle cells in the lesions. There was no significant difference in LRP1 levels when LRP1+/+ vessels were compared with vessels from macLRP1-/-, likely reflecting the abundance of LRP1 in fibroblasts and vascular smooth muscle cells, and the fact that the lesion only occurs in a portion of the entire vessel. Mac-2 immunoblot analysis confirmed the increase in macrophage recruitment into the lesions in both LRP1+/+ and macLRP1-/- mice (Fig. 3h).

### Temporal gene expression patterns during remodeling of carotid arteries reveal enhanced gene expression of *TGF-β2* in macLRP1-/- mice

**Figure 4:**
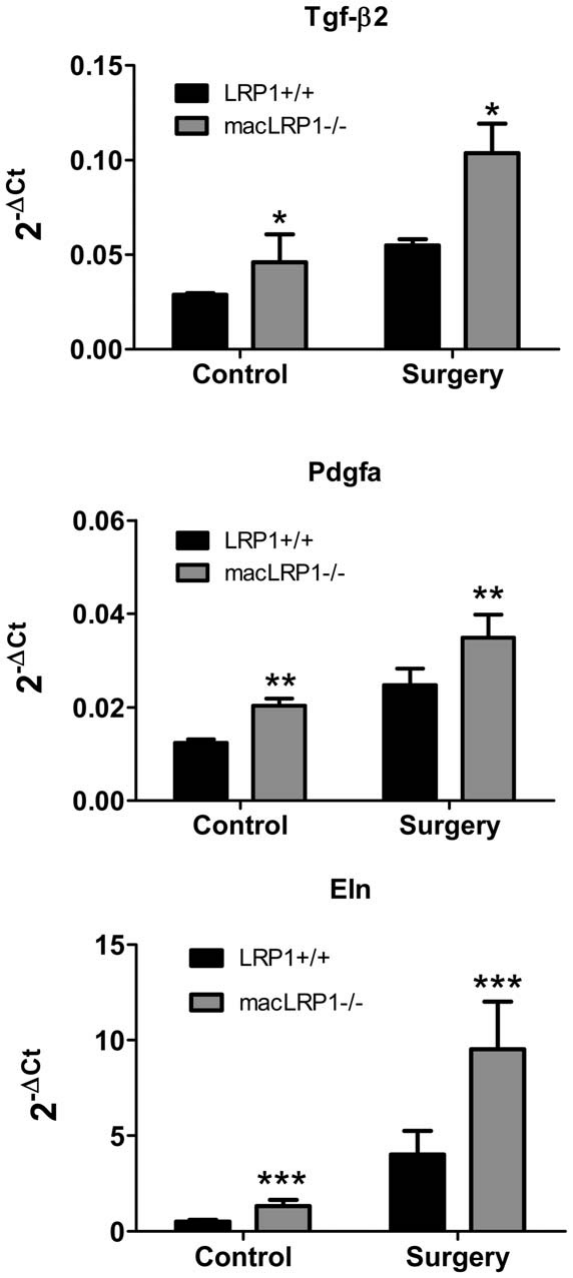
qRT-PCR array analysis identifies increased expression of *Tgf-β2*, *Pdgfa* and *Eln* mRNA in macLRP1-/- vessels. Total RNA was isolated from carotid arteries of control mice (no surgery) or mice undergoing carotid ligation. Carotid arteries from 3–4 mice were pooled to generate a single sample (n) for analysis. qRT-PCR array analysis using the atherosclerosis array from SABiosciences™ was performed. Only 3 of the 84 genes were significantly dependent upon genotype when ligated vessels were compared from LRP1+/+ mice with those from macLRP1-/- mice as assessed by 2-way ANOVA analysis. The relative levels of *TGF-β2* (a), *Pdgfa* (b) and *Eln* (c) are shown for controls; LRP1+/+ (n = 3), macLRP1-/- (n = 3) and for surgery; LRP1+/+ (n = 3), macLRP1-/- (n = 3). Two way ANOVA revealed significant effects for genotype (*p = 0.009; **p = 0.023; ***p = 0.049) and treatment (*p = 0.002; **p = 0.002; ***p = 0.002) with no significant genotype X treatment interaction.

To assess the influence of macrophage LRP1 on events occurring in the vessel wall during vascular remodeling induced by ligation and high fat diet, we employed quantitative RT-PCR arrays to identify changes in mRNA levels of genes involved in this process. As a control for these experiments, we isolated vessels from LRP1+/+ and macLRP1-/- mice that had not undergone ligation. The changes in gene expression were analyzed by a 2-way ANOVA analysis to evaluate the effect of treatment (control vs. ligation) as well as the effect of genotype (WT vs. macLRP1-/-) on the outcome. At day 14 following surgery, the ligated vessels of both LRP1+/+ and macLRP1-/- mice reveal changes in multiple genes encoding extracellular matrix components, pro-inflammatory cytokines and cell cycle related genes whose mRNA levels were altered in response to treatment (Table 1). Importantly, only three genes of 84 tested were identified whose mRNA levels varied as a result of genotype differences (i.e. as a consequence of deletion of LRP1 in macrophages), and these are shown in Figure 4.

**Table 1:** Genes whose expression levels change upon atreatment.

	bFold			bFold	
Symbol	Change	cp	Symbol	Change	cp
Abca1	2.2	0.0426	Lama1	−2.5	0.06
Ace	6.2	0.0427	Msr1	33.2	0.0043
Apoa1	−7.8	<0.0001	Nfkb1	−1.2	0.2865
Apob	7.3	0.003	Npy	−2349.7	0.0019
Bax	5.5	0.0014	Nr1h3	−2.1	0.0116
Bcl2a1a	12.0	0.0027	Pdgfa	2.0	0.0028
Bcl2l1	−2.8	0.0001	Pdgfb	8.3	0.0219
Bid	1.3	0.0233	Pdgfrb	9.6	<0.0001
Ccl2	4.1	0.0249	Ppara	−31.7	0.0019
Ccl5	5.5	0.008	Ppard	3.3	0.0059
Ccr1	5.9	0.0654	Pparg	−12.5	0.0062
Cd44	1.8	0.0051	Ptgs1	−4.2	0.0012
Col3a1	3.2	0.0016	Sele	3.7	0.0099
Ctgf	2.2	0.0245	Sell	30.3	0.002
Cxcl1	8.9	0.0184	Selplg	14.8	0.0002
Eln	7.9	0.0023	Serpinb2	−3.0	0.097
Fabp3	−59.2	0.0009	Serpine1	15.3	0.0755
Fas	−2.0	0.0009	Sod1	−3.3	0.0005
Fn1	41.9	0.0002	Spp1	39.7	<0.0001
Icam1	7.7	0.0004	Tgfb1	7.2	0.0399
Il1a	32.8	0.0025	Tgfb2	1.9	0.0024
Il1b	47.3	0.008	Thbs4	−15.4	<.0001
Il1r2	6.3	0.0041	Tnc	83.2	<0.0001
Il3	244.9	0.0001	Tnf	6.3	0.0047
Il4	−2.1	0.005	Tnfaip3	4.8	0.0053
Il5	−1.9	0.0148	Vcam1	2.9	0.0056
Itga2	1.3	0.1831	Vegfa	−4.1	0.0016
Itgax	143.7	<0.0001	Vwf	3.7	0.0967
Itgb2	7.2	0.0006			

a Treatment refers to carotid ligation surgery.

b WT control mice (i.e. no treatment) vs. WT mice undergoing treatment. The fold change in macLRP1-/- mice with no treatment vs. treatment was very similar, but is not shown.

c 2 way ANOVA, p for control (no treatment) vs. treatment.

One of these genes is the profibrotic gene *TGF­*-*β2,* a member of the TGF-β family. There are three TGF-β family members, TGF-β1, TGF-β2, and TGF-β3, all of which are expressed in the vessel wall and have overlapping functions [19]. Smith et al. [13] demonstrated a prominent role for transforming growth factor–β(TGF-β) in vascular remodeling. During wound healing and tissue repair, TGF-β is known to induce myofibroblastic trans-differentiation [20], SMC proliferation [21], [22] and promote matrix deposition [23]. These events occur via signaling pathways involving the downstream effectors Smad2 and Smad3. A second gene whose expression is enhanced in the macLRP1-/- mice is *Pdgfa*, a growth factor that is known to contribute to vascular remodeling by promoting the proliferation and migration of vascular smooth muscle cells. Expression of the *Pdgfa* gene is induced by low levels of TGF-β [21], [22]. Increased expression of the *Eln* gene may also be a consequence of increased TGF-β2 expression, as activation of the TGF-β signaling pathway induces expression of the *Eln* gene [24]. Interestingly, qRT-PCR analysis of the contralateral vessel reveals no difference in TGF-β2 mRNA levels in LRP1+/+ and macLRP1-/- mice.

**Figure 5:**
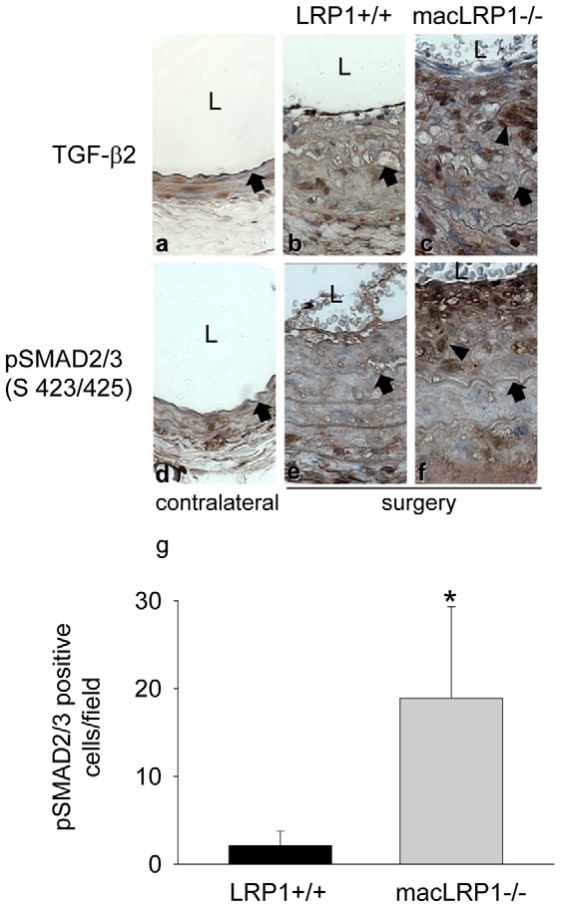
Increased activation of the TGF-β signaling pathway during vessel remodeling in macLRP1-/- mice. Representative images from contralateral (a,d) and ligated vessels (b,c,e,f) stained for TGF-β2 (a,b,c) or phospho-Smad 2/3 (d,e,f). Internal elastic lamina (IEL) is marked by a black arrow. Arrowheads indicate a cell positive for antibody staining while L marks the lumen. (g) five fields from each mouse were assessed for the number of phospho-Smad2/3 positive nuclei for LRP1+/+ (n = 2 mice) and macLRP1-/- (n = 2 mice). *p<0.0001, students *t-*test.

To confirm that TGF-β2 is expressed during vascular remodeling, immunohistochemical analysis was performed on sections of the carotid artery from LRP1+/+ and macLRP1-/- mice. The results, shown in Fig. 5, demonstrate that TGF-β2 is expressed upon carotid ligation, with the majority of TGF-β2 located within the neointima. Further, there appears to be more extensive staining of TGF-β2 in arteries from the macLRP1-/- mice (Fig. 5c).

**Figure 6:**
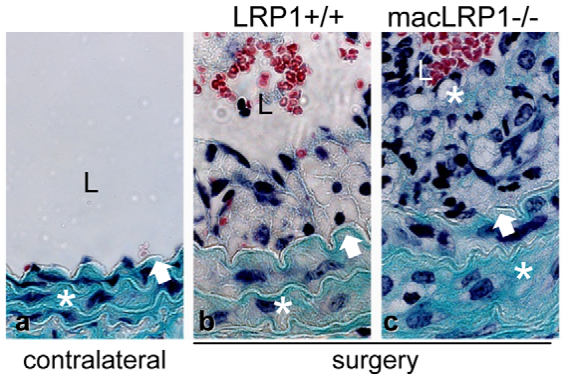
Evaluation of matrix deposition in neointima. Photomicrographs showing the representative Fibrin-Fraser-Lendrum stained sections of contralateral (a) and ligated vessels (b,c). Internal elastic lamina (IEL) is marked by black arrow. White asterisk show collagen matrix specific green color deposition. L identifies the lumen.

To determine if the TGF-β signaling pathway is activated during vascular remodeling, we stained tissue sections with specific antibodies against phosphorylated forms of Smad2/3 (pSmad2/3), which represents activated mediators of transcriptional activation of the TGF-β signaling pathway. pSmad2/3 expression (Fig. 5, d, e, f) was particularly abundant in extensively remodeling areas where neo-intimal thickening occurs. Quantification of the pSmad2/3 positive cell nuclei in these remodeling areas revealed more extensive activation of the TGF-β signaling pathway in macLRP1-/- mice (Fig 5 g). Further, enhanced collagen deposition was noted in vessels from the macLRP1-/- mice using Fibrin-Fraser-Lendrum stained sections (Fig 6).

**Figure 7:**
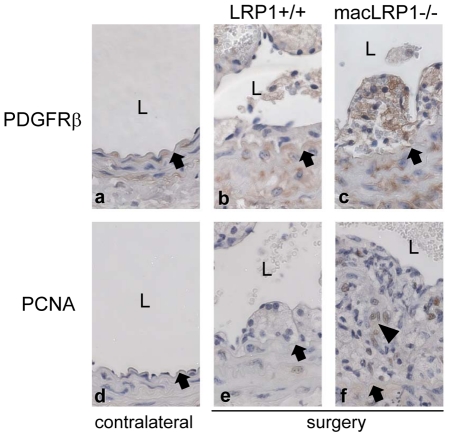
Increased cell proliferation and PDGFR-βexpression during vascular remodeling. Representative anti-PDGFR-β staining (a,b,c) and anti-PCNA staining (d,e,f) for proliferating cells of contralateral (a,d) and ligated carotid arteries (b,c,e,f). Internal elastic lamina (IEL) is marked by a black arrow. Arrowheads indicate the cells positive for antibody staining. L identifies the lumen.

We also examined PDGFRβ expression, and detected increases in this receptor in both medial and neointimal cells (Fig. 7a, b, c) of macLRP1-/- mice. The neointimal cells in both of the remodeled LRP1+/+ and macLRP1-/- carotid arteries were in a proliferative state as shown with proliferating cellular nuclear antigen (PCNA) staining (Fig. 7d, e, f). TUNEL staining did not detect apoptotic cells in the vessel wall (data not shown).

### LRP1 attenuates TGF- β2 mRNA levels in macrophages

**Figure 8:**
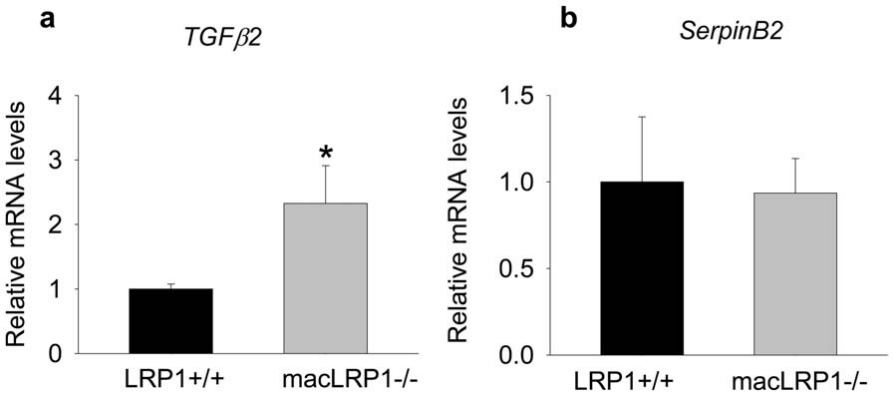
TNF-α attenuates *Tgf-*β*2* expression in LRP1 expressing macrophages. a) Bone marrow derived macrophages were treated with TNF-α overnight. Total mRNA from each sample was isolated and analyzed by qRT-PCR array analysis using the atherosclerosis array from SABiosciences™. The levels of *Tgf-*β*2* (a) and *SerpinB2* (b) are shown for LRP1+/+ and macLRP1-/- cells (n = 3 for each cell type; *p = 0.007, Students t-test).

Macrophages are known to produce TGF-β2, and to determine if the increase in *TGF-*β*2* mRNA in macLRP1-/- mice could result from alterations in gene expression of the macLRP1-/- macrophages, we examined the expression of this gene in bone marrow derived macrophages (BMDM). Since our PCR array data revealed a substantial increase in *TNF-*α mRNA in vessels undergoing ligation (see Table I), we examined the effects of TNF-α on *TGF-*β*2* expression in LRP1+/+ and LRP1-/- BMDM. The results revealed that when BMDM were cultured in the presence of TNF-α, those from macLRP1-/- mice express significantly more mRNA for *TGF-*β*2* than LRP1+/+ macrophages (Fig. 8a). The impact of LRP1 deletion in macrophages appears specific for the *TGF-*β*2* gene, as expression of genes known to be sensitive to TNF-α, such as the *SerpinB2* gene [25], remained unchanged when macLRP1-/- macrophages were compared to those expressing LRP1 (Fig. 8b).

### TGF-β2 induces ERK1/2 and SMAD activation in vascular smooth muscle cells

**Figure 9:**
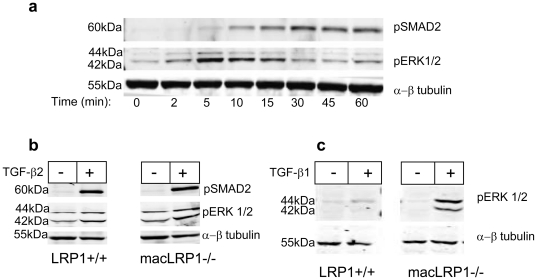
TGF-β2 induces SMAD and MAPK signaling in hAoSMCs and TGF-β-mediated ERK activation is inhibited in LRP1+/+ expressing macrophages. a) hAoSMCs were serum starved for overnight and induced with 2 ng/ml TGF-β2 at indicated times. Cell extracts were analyzed for phospho-SMAD2 (S465/467), phospho-Erk1/2 (T202/Y204) or α/β tubulin for loading control. Results are representative of two independent experiments. b,c) Bone marrow derived macrophages from LRP1+/+ or macLRP1-/- mice were treated with TGF-β2 (b) or TGF-β1 (c). Cell extracts were then analyzed for phospho-Erk1/2 and/or phospho-SMAD2 by immunoblot analysis. α/β tubulin levels represents loading control measured by immunoblot analysis.

While a large number of studies have investigated the effect of TGF-β1 on vascular smooth muscle cells, at present very little is known regarding the potential of TGF-β2 to affect vascular smooth muscle cell function. We therefore investigated the effects of TGF-β2 on the MAPK signaling pathway as well as the canonical TGF-β pathway in vascular smooth muscle cells. The MAPK pathway is of interest, as this pathway is known to play a pivotal role in neointima formation during restenosis [26]. The results, shown in Fig 9a, reveal that TGF-β2 activates both the MAPK pathway as well as the TGF-β pathway as evidence by phosphorylation of ERK1/2 and SMAD 2/3, respectively. We also examined the ability of TGF-β2 to activate the ERK1/2 and SMAD 2/3 pathway in macrophages, and found that TGF-β2 activates both pathways in macrophages, but not in an LRP1-dependent manner (Fig 9b). Interestingly, while we observed a robust ERK1/2 activation when TGF-β1 was incubated with LRP1 deficient macrophages (fig 9c, *right panel*), induction of ERK1/2 activation was attenuated in LRP1+/+ macrophages (Fig. 9c, *left panel*), revealing that LRP1 suppresses TGF-β1 mediated ERK activation.

### LRP1 binds TGF-β2 and regulates the levels of this molecule

**Figure 10:**
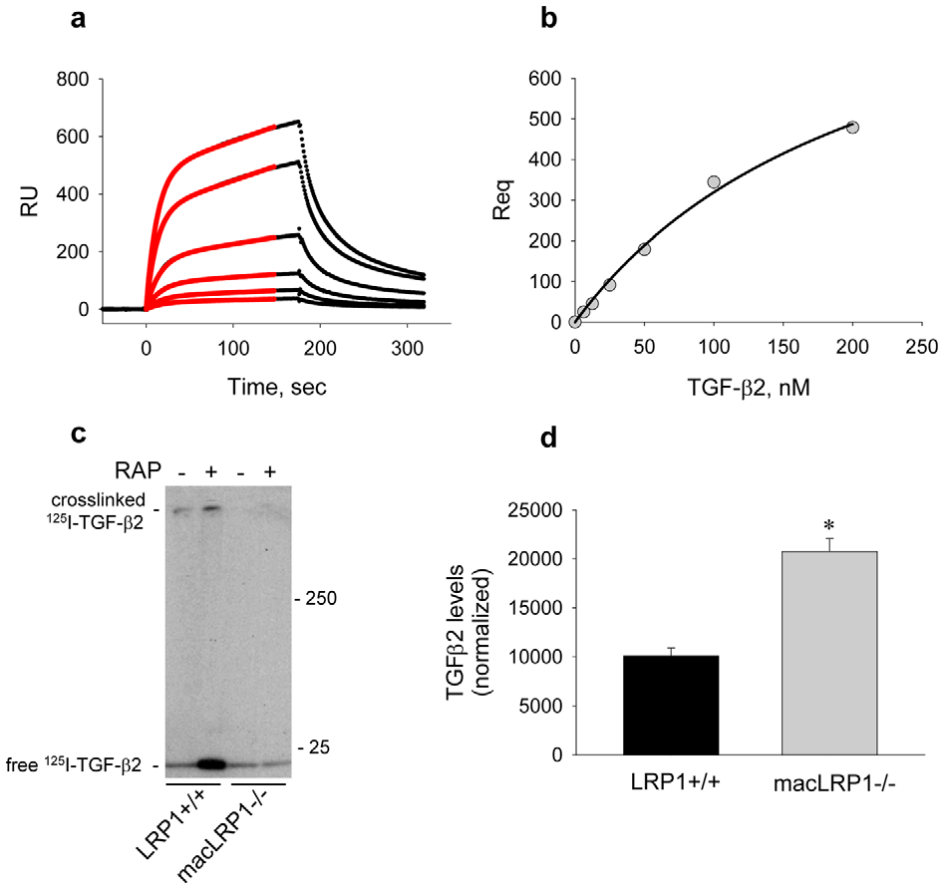
LRP1 binds TGF-β and regulates its levels. a) Surface Plasmon resonance confirms binding of TGF-β2 to LRP1 immobilized on SPR chip. The red line shows the best fit to a pseudo-first order process also containing a non-specific binding component. b) Rmax determined from the fits in (a) are replotted vs TGF-β2 concentrations. The line shows the best fit to a binding isotherm using non-linear regression analysis, revealing a K_D_ of 222 nM. c) TGF-β2 co-immunoprecipitates with LRP1 in LRP1+/+ macrophages. Macrophages from LRP1+/+ or macLRP1-/- mice were incubated with ^125^I- TGF-β2 plus or minus RAP. Following crosslinking, cell extracts were subjected to immunoprecipitation and the immunoprecipitated proteins separated by SDS-PAGE and transferred to nitrocellulose membrane. The membranes were then exposed to autoradiographic film. d) TGF-β2 accumulation in macrophage condition media analyzed by immunoblot analysis. Bone marrow derived macrophages were grown in serum-free media for 72 h. Concentrated culture media (0.5 ml) were subjected to western blot analysis for TGF-β2 expression. Gels were analyzed using NIH ImageJ, and integrated bands were normalized to total cell protein (n = 3 for each cell type, *p = 0.0168, Students t-test).

LRP1 has been suggested to participate as a TGF-β receptor, and crosslinking studies in cells have revealed an association of TGF-β1 with LRP1 [27]. However, it is not known if TGF-β2 is able to bind to LRP1. Thus, to determine if LRP1 is able to directly bind TGF-β2, we conducted surface plasmon resonance experiments with purified components. The results reveal that TGF-β2 binds directly to LRP1 in a dose-dependent manner (Fig. 10a). Estimation of the affinity of LRP1 for TGF-β2 was assessed by quantative analysis of the data, which revealed a K_D_ value of 222 nM. This value is comparable to the affinity (K_D_ = 157 nM) measured for the binding of TGF-β1 to soluble forms of the extracellular domain of the type II TGF-β receptor [28]. To confirm the surface plasmon resonance experiments, we performed co-immunoprecipitation experiments of cell extracts following cross-linking of ^125^I-labeled TGF-β2 to cells. The results of the experiment (Fig. 10c) reveal that ^125^I-labeled TGF-β2 co-immunoprecipitated with LRP1. The specificity of the interaction was confirmed by demonstrating that ^125^I-TGF-β2 was not immunoprecipitated in LRP1-deficient macrophages. Interestingly, RAP was unable to compete for binding suggesting that TGF-β2 binds to a region of LRP1 that is distinct from the LDLa repeats.

The ability of LRP1 to directly bind TGF-β2 suggests that expression of LRP1 might reduce the levels of TGF-β2 due to LRP1-mediated catabolism. To test this, we measured the TGF-β2 antigen level in conditioned media collected from LRP1+/+ and macLRP1-/- macrophages by immunoblot analysis. These studies revealed that the concentration of TGF-β2 in conditioned media from macLRP1-/- cells is more than twice that found in the LRP1 expressing cells (Fig. 10d). Since quantitative RT-PCR revealed that the mRNA levels for TGF-β2 is identifical in resting macrophages from LRP1+/+ and macLRP1-/- mice (data not shown), together the results reveal that LRP1 is also capable of regulating the levels of TGF-β2 protein, most likely by binding and mediating the catabolism of this molecule.

## Discussion

Restenosis is a process that results from accumulation of vascular smooth muscle cells in the intima following vessel injury. It is currently thought that injury induces vascular smooth muscle de-differentiation from a ‘contractile’ phenotype to a ‘synthetic’ phenotype resulting in enhanced proliferation and migration of the de-differentiated cells into the intima [29]. Further, these cells synthesize significant amounts of extracellular matrix proteins. Macrophages accumulate early in the lesions [14], [15] and contribute to vascular remodeling following injury. In the current investigation, we were interested in the contribution of macrophage LRP1 to this process. Our results reveal that LRP1 expression in macrophages minimizes the extent of vascular remodeling in the carotid ligation model, and when LRP1 is genetically deleted in these cells, the formation of the neointima is much more extensive.

To gain insight into the potential mechanisms by which macrophage LRP1 might regulate vascular remodeling, quantitative RT-PCR arrays were employed. These data revealed that *TGF-*β*2* mRNA levels in the vessel wall of macLRP1-/- mice were more than 2-fold higher than LRP1+/+ mice. Immunohistochemical analysis of pSMAD2/3 expression confirmed excessive activation of the TGF-β signaling pathway in the vessels of macLRP1-/- mice. Several experiments confirmed that LRP1 expression in macrophages plays a major role in regulating levels of TGF-β2. First, we demonstrated that treatment of bone marrow derived macrophages with TNF-α leads to a 2-fold increase in the levels of *TGF-*β*2* mRNA in LRP1-deficient cells. Second, we found by employing surface plasmon resonance experiments as well as crosslinking experiments followed by co-immunoprecipitation that LRP1 can directly bind TGF-β2. Third, we showed that macrophages deficient in LRP1 accumulate twice as much TGF-β2 protein in conditioned media when compared with LRP1 expressing macrophages despite the fact that TGF-β2 mRNA is unchanged. Thus it appears that LRP1 regulates TGF-β2 levels by binding this molecule and mediating its catabolism. Based on these data, we propose that the molecular mechanism by which macrophage LRP1 suppresses vascular remodeling is via modulating TGF-β2 expression and by attenuating TGF-β signaling. Upon deletion of LRP1 in macrophages, increased TGF-β2 expression results in increased *Pdgfa* gene expression, which we propose leads to enhanced activation of the PDGF signaling pathway. Increases in *Pdgfa* gene expression in turn may account for increased smooth muscle cell migration and proliferation in the macLRP1-/- mice. It is interesting to note that IL3 levels are extensively increased in both WT and LRP1-deficient mice upon vascular remodeling. IL3 is known to stimulate migration and proliferation of vascular smooth muscle cells [30], and likely contributes to the vascular remodeling in this model.

Several studies support the notion that LRP1 is an important modulator of the TGF-β signaling pathway. Thus, Huang et al. [27] demonstrated that ^125^I-labeled TGF-β could be crosslinked to LRP1, and that this interaction was inhibited by the LRP1 antagonist, receptor associated protein, RAP. These studies also demonstrated that murine fibroblasts in which LRP1 was genetically deleted were not sensitive to growth inhibition by TGF-β [27]. Further, mice with a genetic deletion of LRP1 in vascular smooth muscle cells on an LDLr-deficient background demonstrated nuclear accumulation of phosphorylated Smad2/3 in the aorta [31], revealing that in an atherosclerosis model, smooth muscle cell LRP1 suppresses the TGF-β signaling pathway. The effects of TGF-β on the cells of the vasculature are complex. For example, TGF-β can both inhibit [32] as well as stimulate [21], [33] the growth of vascular smooth muscle cells depending on the conditions. Based on its ability to inhibit vascular smooth muscle cell growth and on its anti-inflammatory activity, it has been suggested that TGF-β plays a protective role in the development of atherosclerosis [34]. However, substantial evidence reveals an important contribution of the TGF-β signaling pathway in restenosis. First, gene transfer of TGF-β into the wall of normal porcine vessels resulted in significant deposition of extracellular matrix accompanied by intimal and medial hyperplasia [35]. Second, transfection of ribozyme oligonucleotides targeted to a common sequence of TGF-β during balloon injury of rat vessels resulted in diminished TGF-β expression and a significant reduction in collagen synthesis and in neointima formation [36]. Third, injection of a recombinant soluble TGF-β receptor II into a rat following balloon injury resulted in a reduction in intimal lesion formation [13]. Taken together, these studies provide compelling evidence that TGF-β participates in vascular remodeling during restenosis. The findings in the current study demonstrate that macrophage LRP1 regulates TGF-β2 levels and attenuates the TGF-β signaling pathway, identifying a new paradigm for regulating vascular remodeling.

In addition to its potential to regulate the TGF-β signaling pathway, LRP1 also modulates other signaling pathways. For example, LRP1 associates with the PDGFR-β [37], [38] and modulates the MAPK and Akt/phosphatidylinositol 3-kinase pathways [39] and PDGF-stimulated vascular smooth muscle cell proliferation *in vivo* [7]. In Schwann cells following peripheral nerve injury, LRP1 functions as a pro-survival receptor, and silencing of Schwann cell LRP1 with siRNA decreases phosphorylated Akt and increases activated caspase-3 [40]. Recent findings reported that LRP1 in thioglycollate-elicited peritoneal macrophages suppressed their inflammatory response to LPS treatment [41]. This was found to occur by a mechanism involving proteolysis of the LRP1 ectodomain, following by subsequent γ-secretase-dependent release of the LRP1 intracellular domain (ICD). The LRP1-ICD was observed to interact with interferon regulatory factor 3 resulting in enhanced nuclear export and degradation. All of these studies, together with the results of the current investigation, highlight the potential of LRP1 to modulate inflammatory events, with the outcome highly dependent upon the initiating stimulus and cellular context.

Within macrophages, LRP1 has been shown to reduce the extent of atherosclerosis in LDL receptor/apoE double knockout mice [8] and in LDL receptor knockout mice [9]. The mechanisms by which this occurs is not understood at this time, but prior work has shown that macrophage migration depends upon LRP1 in coordination with the integrin Mac-1, tissue-type plasminogen activator and its serpin inhibitor, PAI-1 [42], and thus some of the effects may be attributed to increased macrophage retention within the lesion. The results of our current studies further reveal that regulation of the TGF-β signaling pathway may contribute to this effect. However, we need to keep in mind that LRP1 is known to bind over 30 distinct ligands, and thus may protect the vessel wall by a variety of mechanisms, including modulation of signaling pathways as well as via catabolism of various molecules.

In summary, we have demonstrated a protective effect of LRP1 expressed in macrophages on the vessel wall which plays a vital role in reducing neointimal formation thereby preserving vascular function upon vessel wall injury. One of the mechanisms by which this occurs appears to involve regulation of the TGF-β signaling pathway. Future studies with these genetically modified mice will be important for identifying additional mechanisms by which LRP1 protects the vessel wall by preserving lumen diameter and function during restenosis.

## Materials and Methods

### Ethics statement

All animal work in this manuscript was conducted in accordance with the Animal Welfare Act, Public Health Service (PHS) Policy on Humane Care and Use of Laboratory Animals, and the Guide for the Care and Use of Laboratory Animals. All work was reviewed and approved by the University of Maryland Institutional Animal Care and Use Committee. The Animal Welfare Assurance number is: A3200-01, and the protocol number approved is: #0310019, approval date: 3/18/2011.

### Animal Model

LDLr^-/-^ mice were crossbred with mice expressing floxed *loxP* sites flanking the LRP1 gene (both kindly provided by J. Herz, Dallas) as described [43] to generate *LDLR* ^-/-^,*LRP1^flox/flox^* mice. Then *LDLR* ^-/-^,*LRP1^flox/flox^* mice were crossed with mice expressing Cre recombinase, under control of myeloid lineage-specific lysozyme M promoter [44] allowing deletion of the targeted gene in mature macrophages and granulocytes (LysMCre, kindly provided by I. Förster, Munich). Since LRP1 is only expressed in monocytes/macrophages and not granulocytes, this cross generated either macrophage specific LRP1 deletion (macLRP1-/-) or LRP1 wild type mice (LRP1+/+). Mice were weaned at 3 weeks, maintained on a 12-hour light/12-hour dark cycle and fed standard rodent chow (4% wt/wt fat, Harlan Teklad) and water *ad libitum*. Littermate siblings of Cre- (LRP1+/+; *LDLR^-/-^, LRP1^flox/flox^*, *Cre^-/-^*) or Cre+ (macLRP1-/-; *LDLR^-/-^ LRP1^flox/flox^*, *Cre^-/+^*) were used in all experiments. Blood flow cessation model for vessel wall remodeling surgery was performed as described [14]. Following surgery, mice were placed on a “Western” diet (21% wt/wt fat, 0.2% wt/wt cholesterol (Harlan Teklad TD-88137)) for two weeks.

### Quantitative real-time reverse transcriptase (RT)-PCR

Total RNA from snap frozen carotid arteries or cultured BMDMs were isolated using Trizol (Invitrogen) reagent as directed by the manufacturer. The mRNA from 3–4 mice were pooled for each n. In the case of bone marrow derived macrophages, macrophages from 2–3 mice were pooled for total RNA isolation. 1 µg total RNA was used to synthesize cDNA by using the First Strand cDNA Synthesis Kit (SABiosciences). Real-time PCR was performed on an ABI 7900 instrument (Applied Biosystems) by using RT^2^ Real-Time SYBR Green/ROX PCR Master Mix (SABiosciences) and the transcription profile of the Mouse Atherosclerosis RT^2^ *Profiler*™ PCR Arrays (SABiosciences). Data were analyzed based on ΔΔC_t_ fold-change method. *Hsp90ab1* and *Actb* were used as house-keeping genes for data normalization.

### Statistical Analysis

Data are presented as means ± Std and were compared using a 2-way ANOVA test for comparisons of 4 groups and a Student *t*-test for 2 group comparisons. Threshold for significance was set as 1.5 fold change and p≤0.05. All values represent at least 3 independent trials.

### Vessel Morphometry and Immunohistochemistry

Two weeks after inducing arterial injury, animals were anesthetized and perfused with phosphate buffered saline. The entire neck was dissected from each mouse and fixed in 10% buffered formalin. The whole neck was decalcified before embedding in paraffin. Identical whole-neck cross-sections of 5 µm were made from the distal side of the neck beginning at the point of the distally ligated suture until aortic bifurcation. The whole-neck sections were used to evaluate both the injured and the uninjured control vessels on the same section. For each mouse, the apex of lesion was determined by analyzing serial sections at 100-µm intervals for the entire section of the artery. At every 100 µm interval, parallel sections were subjected to routine hematoxylin and eosin (H&E) staining as well as to elastic Van-Gieson (EVG) staining of elastic lamina. In all cases, the apex of the lesion occurred within the same approximate distance from the ligation (0.5 mm). Morphometric measurements were done using ImageJ software on blinded samples. Immunostaining for the following primary antibodies were performed on serial sections at the apex of the lesion: Mac-2 (Cedarlane, Canada), α-SMA (Clone 1A4, Sigma-Aldrich, St Louis, MO), TGF-β2 (Thermo Scientific, Rockford, IL), phospho-Smad2/3 (pSmad2/3) (Ser 423/425) (Santa Cruz Biotechnology, Santa Cruz, CA), PDGFRβ (Chemicon Int.), proliferating cell nuclear antigen (PCNA, Clone PC10, Dako, Carpinteria, CA). Quantification of pSMAD2/3 positive cells was achieved by counting positive nuclei in five random optical fields for two independent remodeling carotid arteries per genotype.

### Surface plasmon resonance

Purified LRP1 was immobilized onto a CM5 sensor chip surface at 5.8 fmol/mm^2^ (3,500 RU) density, by amine coupling in accordance with the manufacturer's instructions (BIAcore AB). One flow cell was activated and blocked with 1 M ethanolamine without any protein and was used as a control surface to normalize SPR signal from receptors immobilized with flow cells. All of the binding experiments were conducted in standard HBS-P buffer, pH 7.4 (BIAcore AB), containing 0.005% Tween 20 and 1 mM CaCl2 at a flow rate of 20 µl/min and temperature of 25°C. Sensor chip surfaces were regenerated by 15s pulses of 100 mM H_3_PO_4_. All injections used the Application Wizard in the automated method. Data were analyzed by fitting to a pseudo-first order process also measuring non-specific binding. The maximum change in response units (Rmax) from this analysis was replotted versus TGF-β2 (Sigma) concentrations (TGF-β2 concentrations were: 6, 12, 15, 50, 100 and 200 nM), and the data were fit to a single class of sites by nonlinear regression analysis using SigmaPlot 11 software.

### Bone Marrow Derived Macrophage Isolation

Bone marrow derived macrophages were generated as described [17]. Briefly, primary monocytes were seeded at 2×10^6^/ 6 mm tissue culture dish and cultured in Bone Marrow Macrophage Growth Medium (BMMGM; DMEM supplemented with 10% FBS, L929 conditioned medium (14% of final volume), penicillin, streptomycin for 7 days. Immunoblot analysis with monoclonal 5A6 antibody [2] confirmed effective deletion of the *LRP1* gene. For signaling experiments cultures were starved in DMEM for overnight and induced with 30 ng/ml TGF-β1 or 2 ng/ml TGF-β2 (Sigma) for 15 min. For qRT-PCR experiments BMD macrophages were starved for overnight and induced with 40 ng/ml recombinant mouse TNF-α (eBioscience). To analyze the TGF-β2 expression and secretion, macrophages were serum starved for 72 hours and conditioned media were collected and centrifuged to clear any cell debris. Protein content of 0.5 ml of conditioned media was concentrated by Strataclean (Stratagen) and analyzed with Western blotting with TGF-β2 antibody. Relative intensities of TGF-β2 were analyzed using ImageJ software.

### Tissue Culture and Immunoblotting

Primary human aortic smooth muscle cells (hAoSMC) were purchased from Clonetics® which were distributed by Lonza (Walkersville, MD) and were cultured according to manufacturer's protocol. All experiments were done between passages number 5 and 8. 70% confluent cells were starved overnight and treated with 2 ng/ml TGF-β2 (Sigma) for indicated times. Cells were washed twice with ice cold PBS and lysates were prepared as described (16). Equal amounts of protein from each lysate were resolved under reducing conditions on 4-20% Tris-Glycine SDS gels. Proteins were transferred to nitrocellulose and membranes were blocked in 3% milk. Membranes were incubated with phospho-SMAD2 (pSMAD2) (S465/467) and phospho-p44/42 MAPK (pERK1/2) (T202/Y204) and αβ-tubulin (Cell Signaling). Antibody binding to the immunoblot was detected by incubation with an appropriate IRDye (LI-COR Biosciences) conjugated secondary antibody. Immunoreactive bands were detected using LI-COR's Odyssey Infrared Imaging System.

### Iodinationation and co-immunoprecipitation of TGF-β2

5 µg of carier-free TGF-β2 (Sigma-Aldrich) was radiolabeled with Na ^125^I (Perkin Elmer) and Chloramine T (Acros Organics) as described [45]. BMD macrophage media were replaced with 1 ml assay buffer (1 mg/ml BSA, 25 mM Hepes pH 8, 1 mM CaCl_2_ in DMEM) containing 17 nM ^125^I- TGF-β2 or 17 nM ^125^I- TGF-β2 and 2 µM RAP and incubated for 2 hrs at 4°C. Cells were washed twice with 2 ml HBS. 1 ml 2 mM 3,3′-Dithiobis[sulfosuccinimidylpropionate] (DTSSP) in HBS was added to the cells and incubated with the cells for 30 min at room temperature. The reaction was stopped by incubating cells with 25 mM TrisHCl (pH 7.5) for 15 min at room temperature. Cells were washed twice with HBS and solubulized in 300 µl RIPA buffer (150 mM NaCl, 10 mM sodium phosphate (pH 7.0), 1 % NP40) with protease inhibitors (complete mini EDTA-free protease inhibitor cocktail, Roche). LRP1 bound protein complexes were immunoprecipitated with 10 µg monoclonal 5A6 antibody and 20 µl Protein G-Dynabead (Invitrogen) overnight at 4°C. Immunoprecipitates were washed twice with RIPA buffer. Supernatants were replaced with 2x non-reducing SDS sample buffer and boiled for 5 min. Extracted protein complexes were separated on 4–12 % gradient polyacrylamide Tris-glycine gel. Gel was transferred to nitrocellulose membrane and exposed to autoradiographic film for 5 days.
